# The effects of calcitonin on post‐orthodontic relapse in rats

**DOI:** 10.1002/cre2.373

**Published:** 2020-12-09

**Authors:** Hussein Abid Ali Muhsin Alnajar, Dheaa H. Al Groosh

**Affiliations:** ^1^ College of Dentistry University of Kufa Kufa Iraq; ^2^ Department of Orthodontics, College of Dentistry University of Baghdad Baghdad Iraq

**Keywords:** calcitonin, orthodontics, rats

## Abstract

**Objectives:**

To determine the effects of systematic calcitonin administration on post‐orthodontic relapse in rat model.

**Material and methods:**

This experimental animal model involved 36 male Wister rats. The maxillary right first molars were moved mesially, using a modified orthodontic appliance delivered 50 gm, for 14 days, retained for 4 days and left to relapse for 10 days. The study group was divided into three subgroups in which a single injection of calcitonin (20 IU/Kg), three injections of calcitonin (20 IU/Kg), each every other day, and normal saline were administered subcutaneously after orthodontic tooth movement has finished. The relapse ratio, histomorphometric analysis including osteoblasts, osteoclasts numbers and bone area and immunohistochemical analysis including the expression of receptor activator of nuclear factor kappa Β (RANK), receptor activator of nuclear factor kappa Β ligand (RANKL) and osteoprotegerin (OPG) were measured and assessed.

**Results:**

The relapse ratio was significantly reduced in the three‐dose calcitonin group (28%) compared to the single dose calcitonin group (34%) and the control group (46%). This was accompanied by a nonsignificant increase in osteoblasts number and bone area in three‐dose calcitonin group and a nonsignificant reduction in osteoclast number. However, the immune histochemical expression of RANK, RANKL and OPG did not show statistically significant difference at the end of relapse period.

**Conclusions:**

Systemic administration of three doses of calcitonin may minimize the relapse ratio in experimentally moved rat molars.

## 1 INTRODUCTION

Relapse after orthodontic treatment is considered as a major problem facing the orthodontists because of the additional cost and time. It was found that only 30%–50% of patients underwent orthodontic treatment maintained a satisfactory alignment after 10 years; whereas after 20 years 10% of them retained a satisfactory alignment (Yu et al., 2013). The etiological factors responsible for the relapse remain unclear and considered multifactorial, however, many causes were proposed such as muscular and soft tissue pressure, stretched gingival and periodontal fibers, changes in tooth inclination and arch dimensions, facial growth, failure to remove the original cause and ongoing bone turnover (Hudson et al., 2012; King et al., 1997; Maleeh et al., 2016). Many attempts were made to minimize or prevent relapse. However, the feasibility that is, patient compliance and technical procedure, and cost effectiveness was halting their success.

During recently, many studies focused on the effective role of calcitonin in the treatment of post‐menopausal osteoporosis. It has been claimed that calcitonin inhibits bone resorption, altering both the number and/or resorptive activity of osteoclasts (Boron & Boulpaep, 2004; Takahashi et al., 2014). Calcitonin is available in many administrative forms, with low price and safe to use. Therefore the current study tried to find a practical, coast effective and simple method to minimize or prevent post orthodontic relapse.

The role of receptor activator of nuclear factor kappa B (RANK), receptor activator of nuclear factor kappa B ligand (RANKL) and osteoprotegerin (OPG) in stimulating the remodeling of bone was elucidated in the past years (Yu et al., 2013). In bone system, the osteoblast cell lineage express RANKL which binds to RANK receptor on osteoclast lineage cells leading to quick differentiation of osteoclast precursors to mature osteoclasts so it has a positive role in osteoclast activation and formation. OPG, on the other hand, is a trap receptor created by osteoblasts which competes with RANKL. It was reported that OPG inhibits osteoclast differentiation in the final stages, induces apoptosis and suppresses the activation of mature osteoclasts. Thus, the balance between OPG production and RANK/RANKL binding controls the bone remodeling (Hudson et al., 2012; King et al., 1997). To the best of our knowledge the systematic administration of calcitonin was not assessed with regard to post orthodontic relapse. The aim of the current study was to determine the effects of systematic calcitonin administration on post‐orthodontic relapse in rat model through assessing the rate of relapse and histomorphometric and immuno‐histochemical analyzes.

## 2 MATERIALS AND METHODS

The study was approved by the scientific research and ethics committee (Ref. 68 in 2015).

### 2.1 Study model design

- Thirty six young adult Wister male rats of 10 weeks of age (weighted 200 ± 20 g) were divided into three groups:

1. Control group: in which rats were injected subcutaneously with normal saline;
2. A single calcitonin (CT) injection group: Rats were injected subcutaneously with 20 IU/Kg of calcitonin (Miacalcin, Novartis, Basel, Switzerland);
3. Multiple calcitonin (CT) injection group: Rats were injected three times subcutaneously with 20 IU/Kg each other day.

The administration of calcitonin and saline was done after orthodontic tooth movement has finished and during retention period.

The rats were acclimated for 2 weeks before starting the experiments in the animal housing at the Higher Institute for the Diagnosis of Infertility and Assisted Reproduction Techniques. The rats were fed pellet diet with tab water ad libitum. The study design includes 14 days of orthodontic tooth movement (OTM) followed by 4 days of retention. At the beginning of retention period, the drug was administered. At day 18 retainers were removed and teeth were allowed to relapse for 10 days, as illustrated in Table 1.

**TABLE 1:** Study model protocol

Time	Action
Day 1	Impression. Orthodontic appliance fitting and starting tooth movement.
Day 15	Impression. Retention period using ligature wire. Drugs injection.
Day 17	Second CT injection for multiple CT group.
Day 19	Third CT injection for multiple CT group. Retainers removal for all groups
Day 29	Impression. Animal sacrifice.

### 2.2 Orthodontic appliance delivery

**FIGURE 1:**
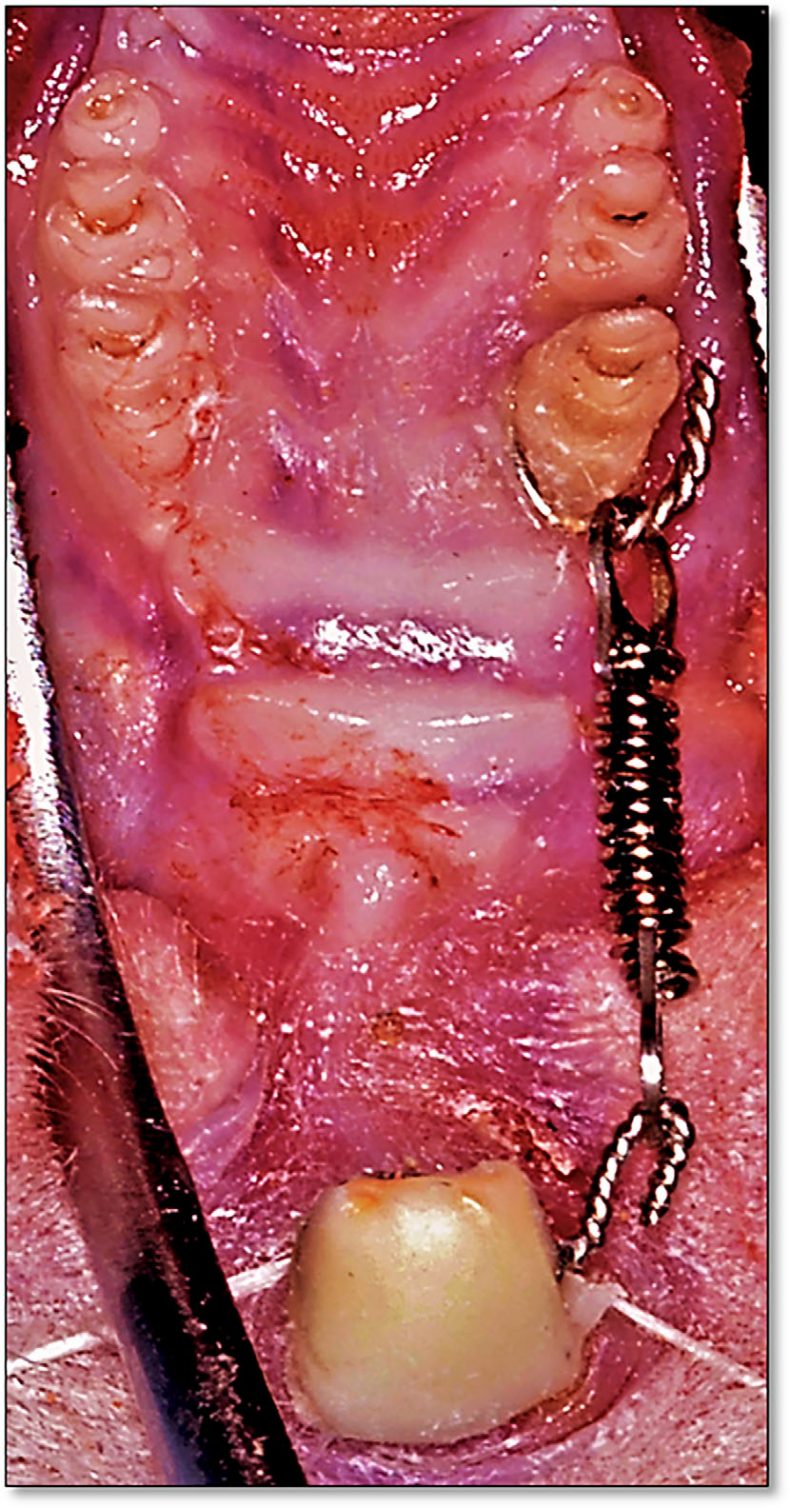
Orthodontic appliance fitted in rat's mouth

Orthodontic appliance was modified according to Yadav et al. (2015) and Franzen et al. (2015) The rats were anesthetized with an intramuscular injection of Ketamine (87 mg/Kg) (Ketamine 10%, Alfasan, Woerden, Holland) and Xylazine muscle relaxant (10 mg/Kg) (XYL‐M2 injectable solution 25 ml, VMD, Arendonk, Belgium). The two drugs were mixed in a ratio of 2:1 (Ketamine: Xylazine). A custom made holding rack was fabricated from a wooden plate to which four metal screws were secured. The mouth of the rat was propped using metal ring that attached to the lower incisors and cotton thread was attached to the upper incisors. An orthodontic ligature wire (Ligature Wire Spool 0.010, DCA, West Chester, USA) was passed interdentally between the first and second right maxillary molars and ligated around the first maxillary molar. The wire was imbedded within a small groove prepared on the mesial surface of the tooth near the gingival margin to prevent its dislodgement and the excess of the wire was cut to leave 4–5 mm for the attachment of low force/deflection rate nickel–titanium coil spring (Rematitan “LITE” tension springs, Dentaurum, Ispringen, Germany) that delivered 50 g of force. The other end of the spring was attached to another ligature wire ligated around the maxillary incisors. Similarly, grooves on the facial, distal and lingual surfaces of the maxillary central incisors were prepared to prevent the dislodgement of the ligature wire. Acid etching (Meta etchant, Meta Biomed, Chungbuk, Korea) was applied to the maxillary incisors followed by the application of bonding adhesive material (Ortho Solo, Ormco, CA, USA) then a small amount of light cured composite (Enlight, Ormco, CA, USA) was applied and adapted to the labial and palatal surfaces of the maxillary incisors to cover the ligature wire and aid in its retention (Figure 1).

### 2.3 Relapse ratio measurement

The impression procedure was done using low viscosity Vinyl Polysiloxane wash material (Imprint II VPS Impression Material, 3 M ESPE, Irvine, USA) with mixing and intraoral tips (Fernández‐González et al., 2016; Miller et al., 2007). The light body impression material was injected around the maxillary molars with the aid of a cement spatula (Cement spatula, Sklar, West Chester, USA) that was used to reflect the cheeks and provide a clear and accurate impression. The impression material was left for 4 min to set, according to the manufacturer's instructions, before being removed from the mouth. All the impressions were vacuumed poured with type IV stone (Elite Rock, Zhermack, Badia Polesine, Italy) using a vibrator to minimize air bubbles entrapment. After setting, the models were trimmed to make the occlusal surface parallel to the model base using a spirit level (Al‐Duliamy, 2011). Casts were then photographed using digital camera (Nickon.D7100, Nickon, Tokyo, Japan) supported with a macro lens (105 mm F2.8 EX DG OS HSM Macro, Sigma, Kanagawa, Japan) and a ring flash (Electronic flash macro EM‐140 DG, Sigma, Kanagawa, Japan) (Miller et al., 2007). The photos were taken perpendicular to the occlusal surface and a ruler was placed adjacent to the models to identify the magnification factor (Adachi et al., 1994; Igarashi et al., 1994).

The movement and relapse rate of the maxillary right first molar was measured relative to the third molar in order to decrease any influencing error resulting from the growth of maxilla or other physiological changes. Molar movement was measured from the distal end of the maxillary third molar to the distal groove of the maxillary first molar (Hudson et al., 2012) using image J software (Miller et al., 2007) as follow:

TM=D2−D1,

AR=D2−D3

RR=AR/TM×100%.

where D1 is the distance between the distal end of upper third molar to the distal groove of the first molar before orthodontic treatment and measured in mm to the nearest 0.01 mm. D2 is the distance between the same reference landmarks after 2 weeks of OTM. D3 is the distance between the same reference landmarks after 2 weeks of appliance removal. TM is the amount of OTM. AR is the amount of relapse. RR is the ratio of relapse.

### 2.4 Tissue preparation

After scarifying the animals, the whole maxilla was separated from the body and placed in 10% natural buffered formalin for one night. Then the specimens were rinsed with water and placed in 10% Ethylene diamine tetra acidic acid solution (EDTA, Sigma–Aldrich, Missouri, United States) (PH = 7) for decalcification for about 3 weeks (Sarsfield et al., 2000). The solution was changed every other day and the bone was probed with a needle to verify its texture for sectioning. After complete decalcification, the right half of each maxilla was oriented in the center of paraffin block to make the microtome sectioning blades passed parallel to the long axis of the upper molars. This allows obtaining longitudinal sections of the teeth and their surrounding bone and periodontal ligament (PDL).

### 2.5 Histological analysis

From each animal, two serial longitudinal sections were taken through the mesial root of the maxillary first molar and stained with Hematoxylin and Eosin (H&E) stain (Sigma–Aldrich) to perform the hostological and histomorphometric analysis for the tension and pressure sides of the mesial root (Heller & Nanda, 1979; Manrique et al., 2015; Ong et al., 2000).

Tissue sections were deparaffinized two times in xylene. Then rehydrated by immersion in absolute alcohol. After that, the sections were placed in 95% alcohol followed by 70% alcohol. Tissue sections were stained by placing them in Hematoxylin for 8 mins. The reverse side of the slides washed for 5 mins in running tap water, then differentiated in 1% acid alcohol. The sections were blued in saturated lithium carbonate solution (Sigma–Aldrich) for up to 1 min before washing in running tap water for 5 mins. After that, the slides were dipped in 95% alcohol for 10 times and counterstained for less than 1 min in Eosin B solution. The slides were dehydrated with 95% alcohol then with absolute alcohol before being placed in two changes of xylene. Finally, the slides were mounted. This procedure followed Ihcworld, H&E staining method and protocol.

**FIGURE 2:**
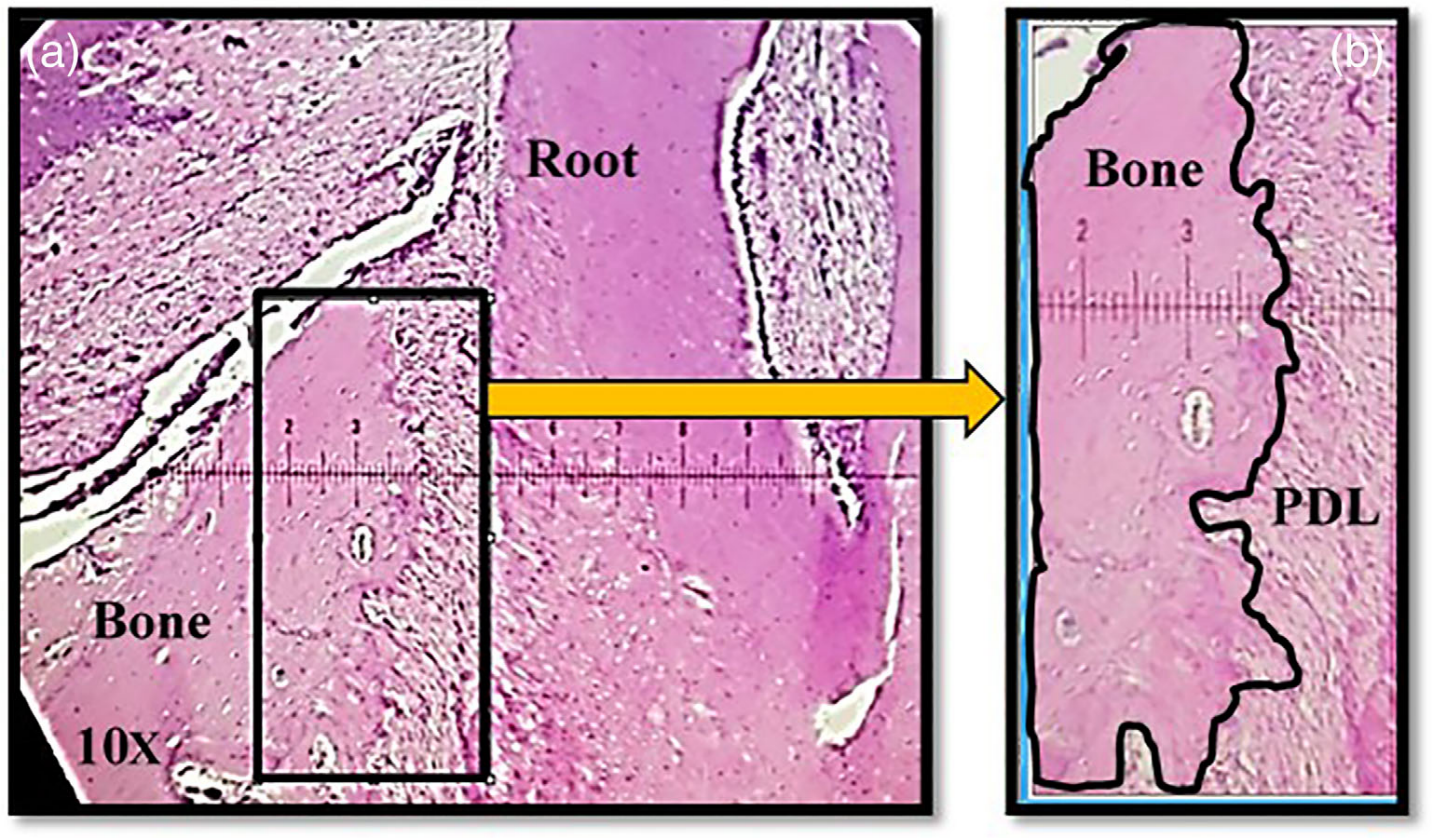
Bone area measurement. (a) The black rectangle represent the selected field adjacent to pressure side. (b) The black line refers to the correspondent bone area

The first molar were examined histologically by an investigator blinded test with a histopathology specialist (Hudson et al., 2012). Two photographs were taken to the mesial root sections; one for the pressure side and the other for the tension side. During imaging procedure, a graduated eye piece was used to aid in positioning of the root to the center of the field. Two areas of each section were examined for alveolar bone response and these standardized areas were outlined by a framed field using the image J software (Figure 2) (Araújo et al., 2015; Heller & Nanda, 1979; Hudson et al., 2012; Shirazi et al., 2002).

#### 2.5.1 Osteoclast and osteoblast cells counting

**FIGURE 3:**
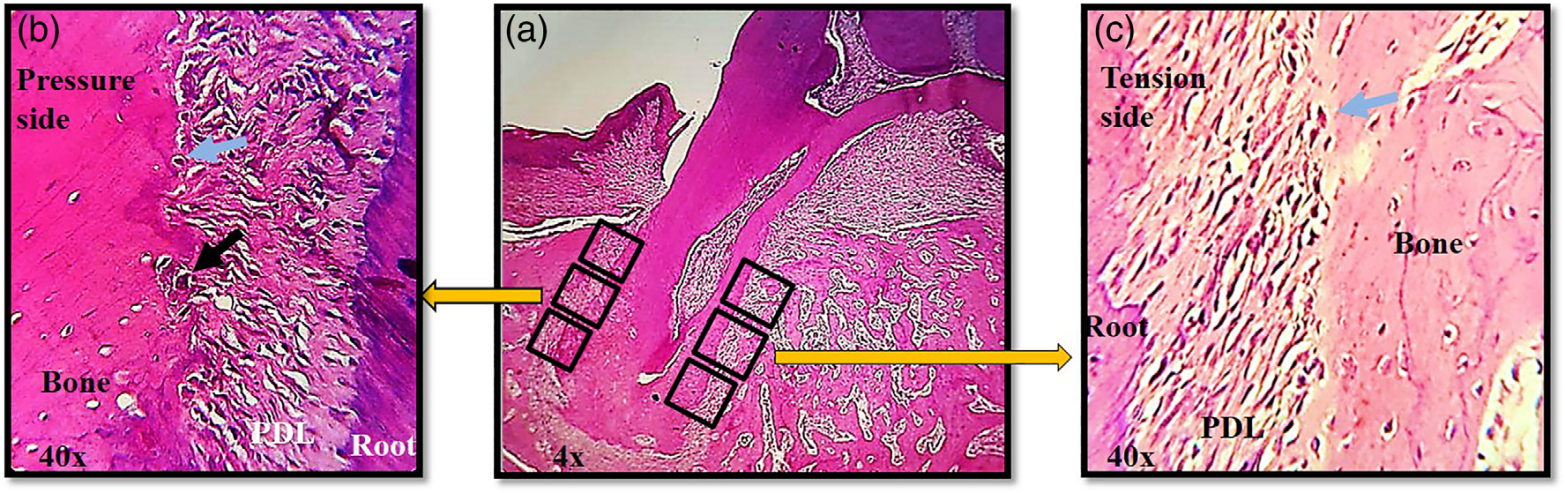
Histopathological section. The highlighted fields in (a) showed the alveolar bone on the pressure (b) and tension (c) sides. The blue arrows show the osteoblast cells in osteoid tissue and the black arrow shows an osteoclast cell

Osteoclast cells were detected adjacent to the alveolar bone surface. These cells were large in size, stained with eosin and has large multiple rounded nuclei usually more than three and located in a resorption cavity called the Howships lacuna. While the osteoblasts were located on the surface of osteoid or bone and they were cuboidal with a plump configuration and big nuclei (Ghajar et al., 2013). Cells were counted directly in both pressure and tension sides of the mesial root of the maxillary first molar using light microscope with ×40 magnification power. The pressure and tension sides were divided into fields with the aid of graduated eye piece. In each field the osteoblasts and osteoclasts were counted directly then the average of all fields in the pressure or tension sides was taken (Figure 3) (Araújo et al., 2015; Haq et al., 2016; Heller & Nanda, 1979; Hudson et al., 2012; Johnson, 2007; Ong et al., 2000).

#### 2.5.2 Histomorphometric analysis

Two areas on pressure and tension sides were selected to measure the total bone area near the mesial root; images were analyzed using image J software (Salazar et al., 2015).

### 2.6 Immuno‐histochemical analysis (RANK, RANKL and OPG expression)

**FIGURE 4:**
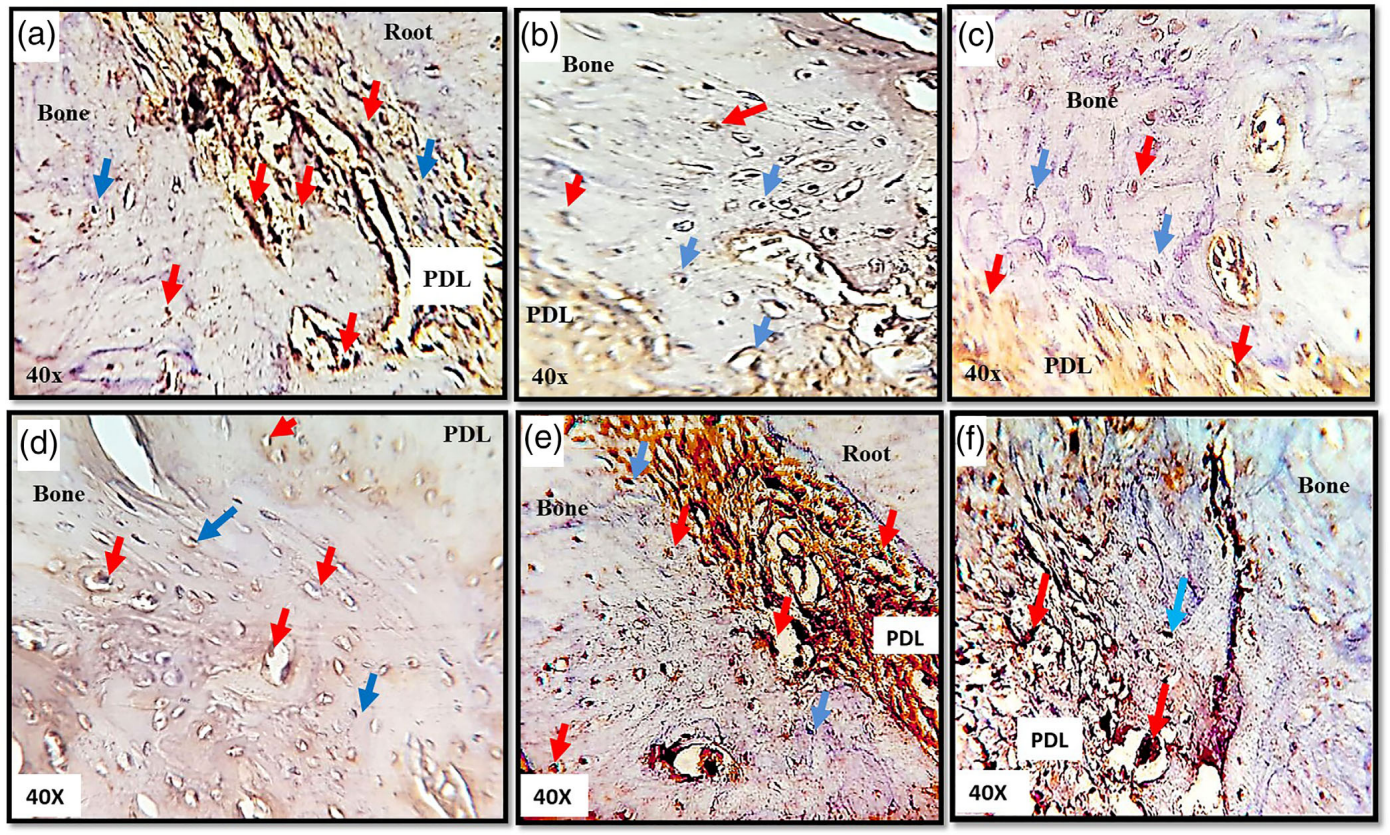
Immunohistochemistry sections in tension sides. (a–c) RANK, RANKL and OPG expression in control group respectively. (d, e) RANK and RANKL expressions in single calcitonin group. (f) OPG expression in multiple calcitonin group. Red arrows refer to the positive expression and the blue arrows refer to the negative expression

Immuno‐histochemical staining was done using an immuno‐enzymatic antigen detection system (ab80436 – EXPOSE Mouse and Rabbit Specific HRP/DA) and RANK, RANKL, OPG primary antibodies (Biorbyt, Cambridge, UK). The pressure and tension sides were divided into fields, and the cells that shows a positive immuno‐histochemical expression of RANK, RANKL or OPG was demonstrated by the presence of brown granular DAB (3,3′‐diaminobenzidine) staining pattern within the cells and tissues of the alveolar bone and periodontal ligament. The negative cells, on the other hand, were bluish in color. The percentage of RANK, RANKL and OPG positive cells (osteoblasts, osteoclasts, fibroblasts, osteocytes) in PDL and alveolar bone in each field was calculated under light microscope with magnification power of ×40 by dividing the number of the mentioned markers +ve cells to the total number of cells (positive and negative) in all fields of interest in either pressure and tension sides; the average of all fields were taken (Figure 4) (Han et al., 2010; Robling et al., 2008; Sanmuganathan, 2011).

Then the averages of percentages were catagorized into scores as follow:

0: <5% (Negative expression);

1:5%–30% (Weak expression);

2:31%–50% (Moderate expression);

3:51%–75% (Strong expression);

4: >75%. (Very strong expression) (Loreto et al., 2013)

### 2.7 Statistical analysis

The normality of data was checked using the Q‐Q plot and box‐plot (Burdenski, 2000; Korkmaz et al., 2014). Descriptive statistics were performed to all variables. A Kruskal–Wallis test was used to compare between the groups; if there was a significant difference a Mann–Whitney *U* test was used to compare between the experimental groups and the control group separately (Richardson, 2010).

## 3 RESULTS

### 3.1 Relapse ratio

**FIGURE 5:**
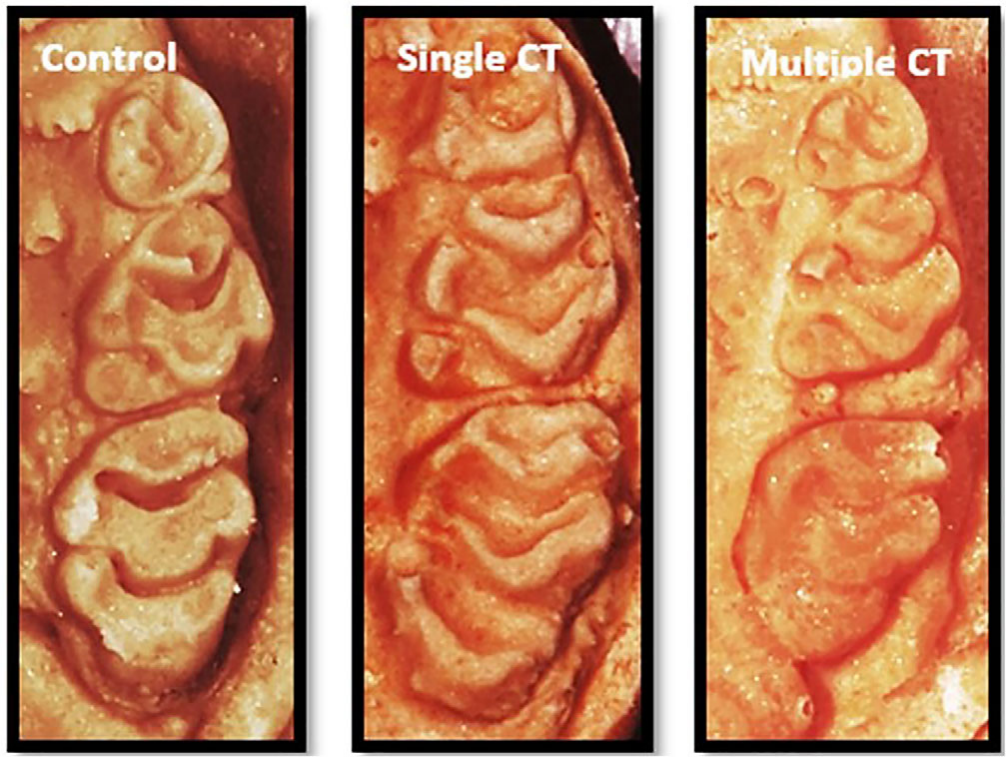
Interdental space after tooth movement between the first molar and second molar at the end of the study period for the three groups

Kruskal–Wallis and Mann–Whitney tests were performed for the study groups and showed that there was a significant difference between the control group and the multiple calcitonin injection group (Tables 2 and 3). Figure 5 shows the difference in the interdental space after relapse period for the three groups.

**TABLE 2:** Descriptive statistics and Kruskal–Wallis test of the relapse ratio (*N* = 13)

Groups	Minimum (%)	Maximum (%)	Mean (%)	SD (%)	Median (%)	Kruskal–Wallis test (*p* value)
Control	21	75	46	16	46	.024*
Single calcitonin	10	48	36	12	37	
Multiple calcitonin	14	51	28	10	27.5	

^*^ *p* < .05 means significant.

**TABLE 3:** Mann–Whitney test for the relapse ratio between the experimental and control groups

Groups	*N*	Mean rank	Sum of ranks	Mann–Whitney (*U*)	*p* value
Control	13	11.3	113	48.000	.287*
Single calcitonin	13	8.56	77		
Control	13	13.85	138.5	32.000	.011**
Multiple calcitonin	13	7.15	71.5		

^*^ *p* > .05 means nonsignificant.

^**^ *p* < .05 means significant.

### 3.2 Osteoblasts and osteoclasts numbers

**FIGURE 6:**
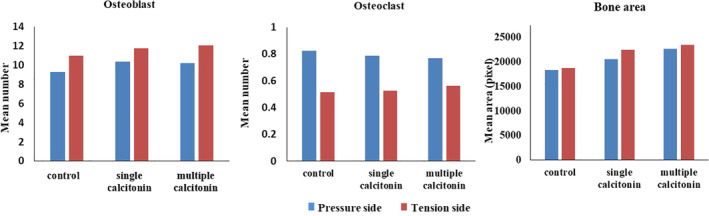
Histogram representation of histomorphometric analysis for pressure and tension sides for the osteoblast cells, osteoclast cells and bone area (in pixel); *N* = 13

Although there was an increase in the mean number of osteoblasts following the single and multiple CT administration and a reduction in the mean number of osteoclasts in the single and multiple CT groups (Figure 6), there was no statistically significant difference among the groups in the pressure and tension sides as revealed by Kruskal–Wallis test (*p* = .339, .147 for osteoblast in pressure and tension sides respectively; and *p* = .874, .773 for osteoclast in pressure and tension sides respectively).

### 3.3 Bone area

There was no significant difference in bone area in single and multiple calcitonin groups regardless of the examined fields (*p* = .527 and .165 for pressure and tension sides respectively) (Figure 6).

### 3.4 Immuno‐histochemical expression

**FIGURE 7:**
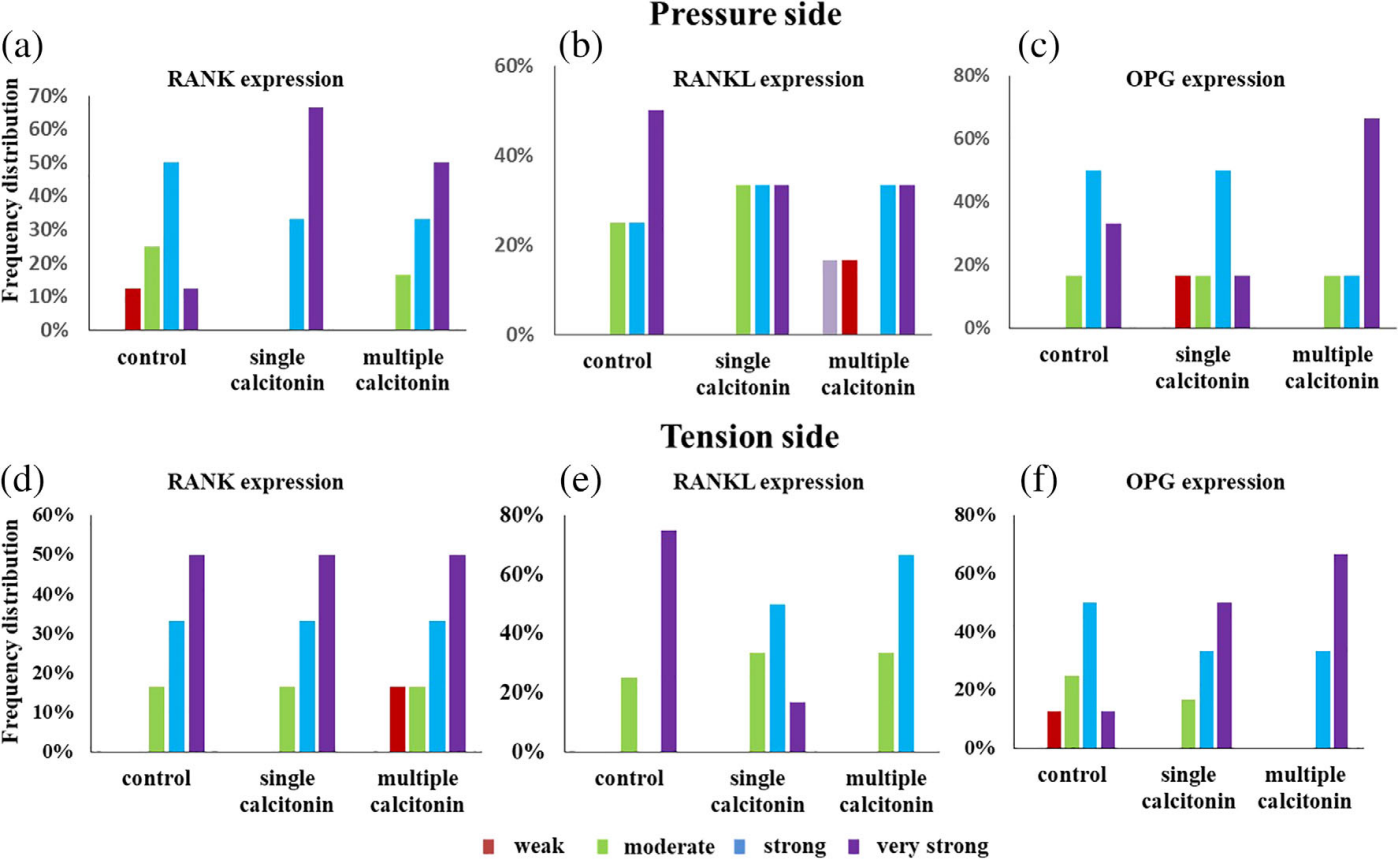
Histogram representation of immunohistochemistry analysis. (a–c) Frequency distribution of RANK, RANKL and OPG expressions in pressure side. (d–f) Frequency distribution of RANK, RANKL and OPG expressions in tension side (*N* = 13)

Figure 7 shows a non‐ significant difference in the percentage of RANK, RANKL and OPG expression between the experimental groups (*p* = .270, .371, .087 and *p* = .131, .304, .927 for pressure and tension sides respectively).

## 4 DISCUSSION

It has been claimed that calcitonin, a thyroid hormone, reduces the blood calcium, inhibits bone resorption, alters both the number and/or resorptive activity of osteoclasts (Boron & Boulpaep, 2004). To the best of the authors' knowledge, calcitonin administration after OTM has not been investigated with regard to minimizing post‐orthodontic relapse.

Optimizing drug dose and drug administration frequency was obtained according previous studies (Al Najar & Al Groosh, 2017; Laitinen et al., 1992). The appropriate calcitonin dose was set to 20 IU/kg. Laitinen et al. (1992) found that the calcitonin dose threshold for food intake in rats was 20 IU/kg and had the strongest effect at 40 IU/Kg especially when given frequently for 10 days. Additionally, they reported a dose dependent reduction in body weight during high dose of CT treatment periods. Furthermore, Kavuncu et al. (2003) found that 20 IU/Kg of CT did not affect the secretion of parathyroid hormone which is a strong bone anabolic hormone; moreover, the bone mineral density and trabecular bone volume were preserved when this dose was administrated in ovariectomized rats.

In the current study, optimizing the CT was carried out through the administration frequency as reflected by the clinical and histological findings of the pilot study. With three‐dose administration of calcitonin with 20 IU/kg a reduction in the amount of relapse occurred without adverse effect on the macroscopical parameter that is, body weight and animal wellbeing (data not included).

The results showed a statistically significant reduction in the relapse ratio in multiple CT group. This could be due to inhibition of bone resorption as a result of reduction in the number of osteoclasts following CT administration which was supported by the findings of Lymperi et al. (2011). Furthermore calcitonin may inhibit bone resorption by decreasing the activity of osteoclasts by decreasing their ruffled border (Diravidamani et al., 2012). This was reflected through microscopical images which showed that their number was reduced in the single and multiple CT groups; additionally, the OPG expression showed a high percentage in both CT groups. However, these expressions were statistically not significant which could be due to the quick onset or short term inhibitory effect of CT on bone resorption as reported to lasted for 8–16 hrs (Zikan & Stepan, 2002). Another explanation could be that the number of osteoclasts was stabilized after the 14 days of relapse “as designed in the current study” and bone remodeling at the experimental side was almost similar to that of the control side (Franzen et al., 2013; King et al., 1997).

Additionally, a reduction in the relapse ratio following CT administration could be explained by its ability to increase the synthesis of collagen that aided in tissue repair after the onset periodontitis (Sondergaard et al., 2010; Wei et al., 2017). The synthesis and remodeling of collagen fibers and periodontal tissues turnover considered as an important factors during the retention period (Tenshin et al., 1995; Yadav et al., 2015).

In addition to remodeling of periodontal tissues, CT may minimize the relapse by increasing the bone mineral density which was found to be increased following CT administration (Mehta et al., 2003; Muñoz‐Torres et al., 2004; Wang et al., 2013) or by increasing the quantity of bone which was reflected in the multiple CT group. This was supported by the findings of Weiss et al. (1981) and Gruber et al. (1984). In addition to its stimulatory effect on osteoblasts which was found to be increased in CT groups.

In conclusion, multiple administration of calcitonin systematically reduced the relapse ratio significantly in rat's model. Although it caused a nonsignificant difference in osteoblast and osteoclast cells numbers, it may enhance bone activity by reducing the RANKL and increasing the OPG expressions. However, further investigations were needed to confirm the inhibitory effect of calcitonin on bone resorption at an earlier period of administration.

## AUTHOR CONTRIBUTIONS

Hussein Abid Ali Muhsin Alnajar: Data curation, Roles/Writing—original draft, Funding, Investigation, Software, Formal analysis, Visualization and Validation. Dheaa H. Al‐Groosh: Supervision, Conceptualization, Resources, Writing—review and editing, Funding, Project administration, Methodology, Visualization and Validation.

## Data Availability

Data subject to third party restrictions
